# Supplementary material for: Rapid in vitro quantification of TDP-43 and FUS mislocalisation for screening of gene variants implicated in frontotemporal dementia and amyotrophic lateral sclerosis
Source: Sci Rep. 2021 Jul 21;11:14881. doi: 10.1038/s41598-021-94225-1 (PMC8295343; doi:10.1038/s41598-021-94225-1)
Supplement: Supplementary file 1 — Supplementary Figures. [file 41598_2021_94225_MOESM1_ESM.pdf]

Rapid *in vitro* quantification of TDP-43 and FUS mislocalisation for screening of gene variants implicated in frontotemporal dementia and amyotrophic lateral sclerosis

Lisa J. Oyston[1], Stephanie Ubiparipovic[1,2], Lauren Fitzpatrick[1], Marianne Hallupp[1], Lauren M. Boccanfuso[1], John B. Kwok[1], Carol Dobson-Stone[1*]

[1]Brain and Mind Centre and School of Medical Sciences, Faculty of Medicine and Health, The University of Sydney, Camperdown, NSW 2006, Australia

[2]Garvan Institute of Medical Research, Darlinghurst, NSW 2010, Australia

Supplementary Figures

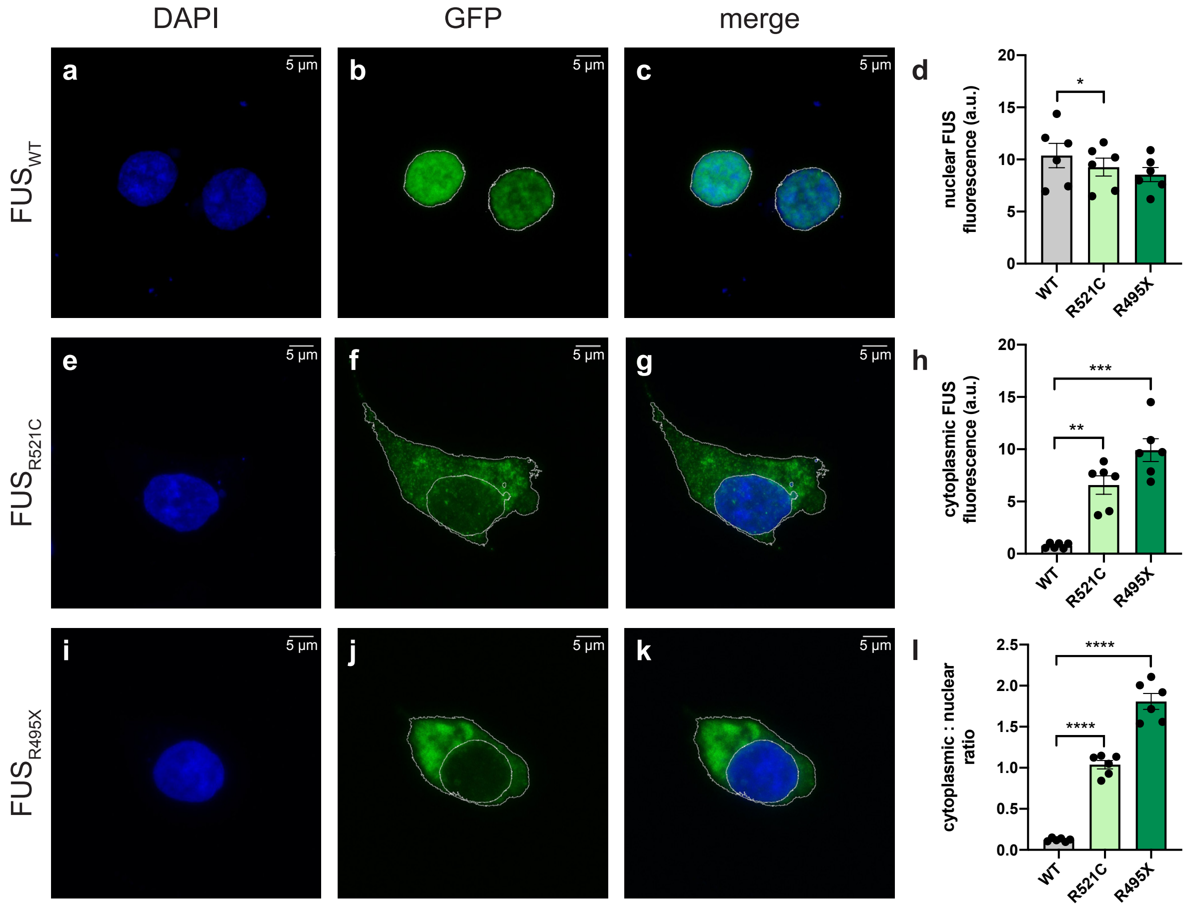

Supplementary Figure S1. Detection of FUS cytoplasmic mislocalisation with exogenous expression of *FUS* mutations. Representative images of HEK293 cells overexpressing GFP-tagged (**a-c**) FUS_{WT}, (**e-g**) FUS_{R521C} or (**i-k**) FUS_{R495X}. Nuclei were visualised with DAPI (blue). (**d**) Quantification of the fluorescence intensity of the nucleus shows a small decrease in nuclear FUS expression in FUS_{R521C}-expressing cells. (**h**) Quantification of the fluorescence intensity of cytoplasmic FUS shows significantly higher cytoplasmic FUS expression in FUS_{R521C} and FUS_{R495X}, when compared to FUS_{WT}. (**l**) Quantification of the cytoplasmic/nuclear ratio of exogenous FUS shows a marked increase in FUS_{R521C}- and FUS_{R495X}-expressing cells when compared to FUS_{WT}. Scale bars = 5 µm. Data is represented as mean ± SEM. a.u. = arbitrary units. *p<0.05; **p<0.01; ***p<0.001; ****p<0.0001.

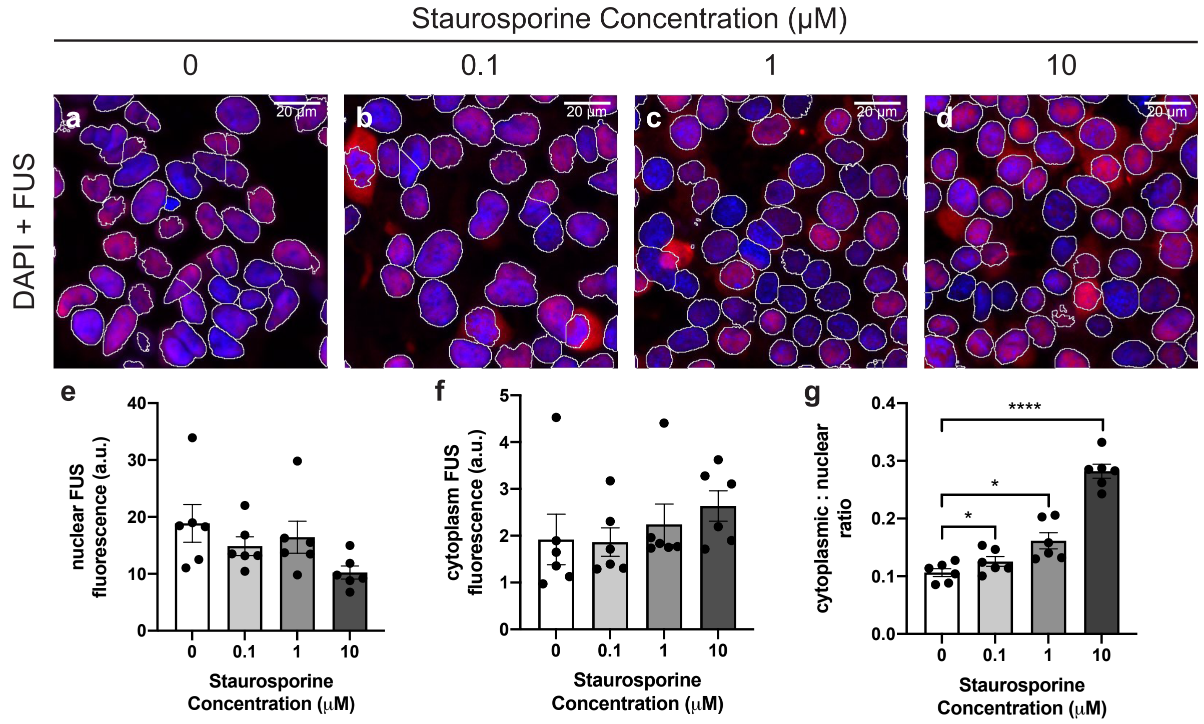

Supplementary Figure S2. Detection of endogenous FUS cytoplasmic mislocalisation following staurosporine treatment. (**a-d**) Representative fluorescence images of HEK293 cells treated with increasing concentrations of staurosporine. FUS was dectected by immunofluorescent staining (red) and nuclei were visualised with DAPI (blue). (**e**) Quantification of nuclear FUS fluorescence intensity and (**f**) cytoplasmic FUS fluorescence intensity showed no difference between groups. (**g**) Quantification of the FUS cytoplasmic/nuclear ratio showed an increase in all treatment groups when compared to those treated with DMSO alone. Scale bars = 20 μm. Data is represented as mean ± SEM. a.u. = arbitary units. *p<0.05; ****p<0.0001.

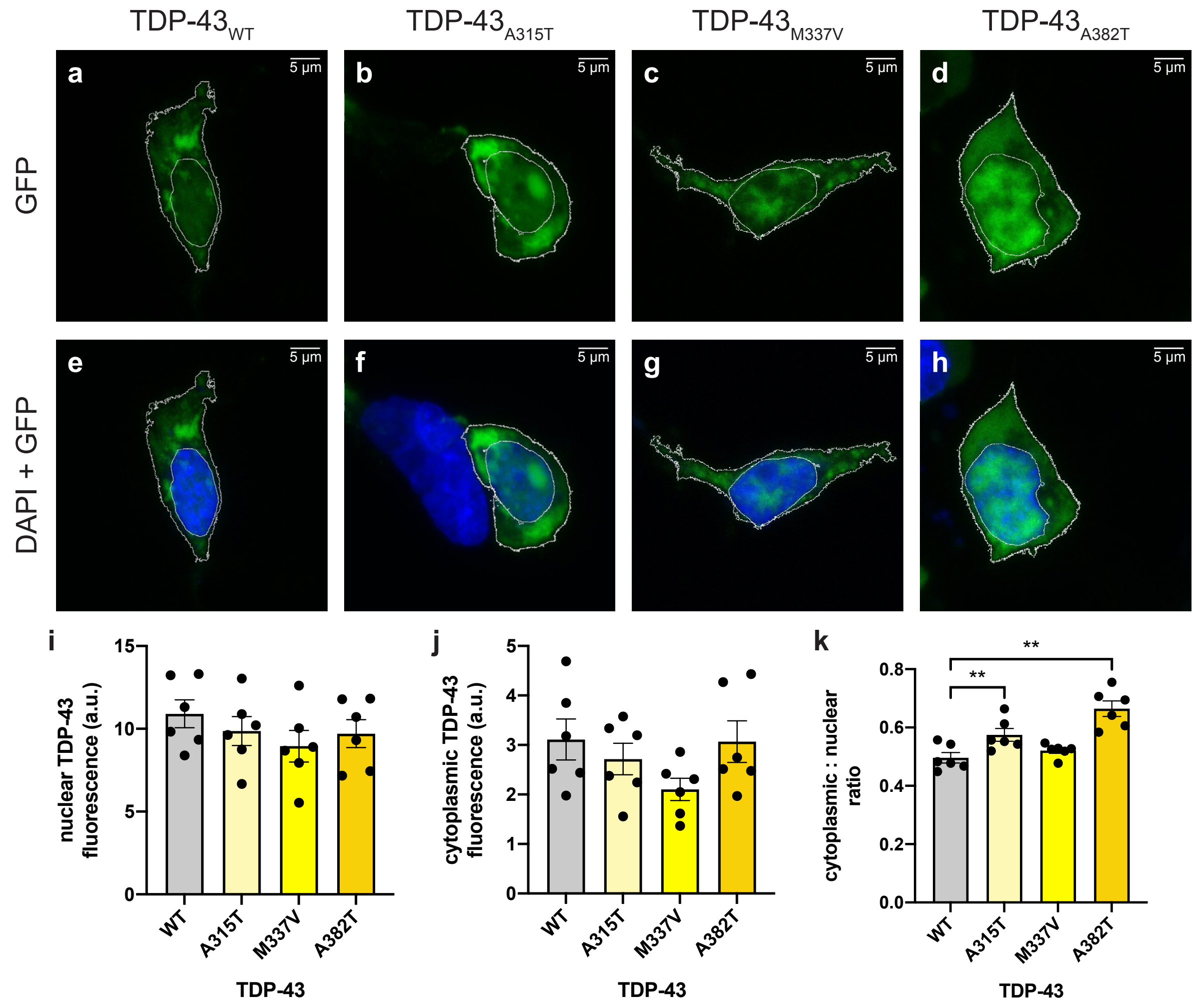

Supplementary Figure S3. Detection of TDP-43 cytoplasmic mislocalisation with exogenous expression of *TARDBP* mutations. Representative images of HEK293 cells overexpressing GFP-tagged (**a, e**) TDP-43$_{WT}$, (**b, f**) TDP-43$_{A315T}$, (**c, g**) TDP-43$_{M337V}$ or (**d, h**) TDP-43$_{A382T}$. Nuclei were visualised with DAPI (blue). (**i**) Quantification of the fluorescence intensity of the nucleus and (**j**) the cytoplasm shows no difference in TDP-43 expression. (**k**) Quantification of the cytoplasmic/nuclear ratio of exogenous TDP-43 shows an increase in TDP-43$_{A315T}$ and TDP-43$_{A382T}$-expressing cells, when compared to TDP-43$_{WT}$. Scale bars = 5 μm. Data is represented as mean ± SEM. a.u. = arbitrary units. **$p<0.01$.

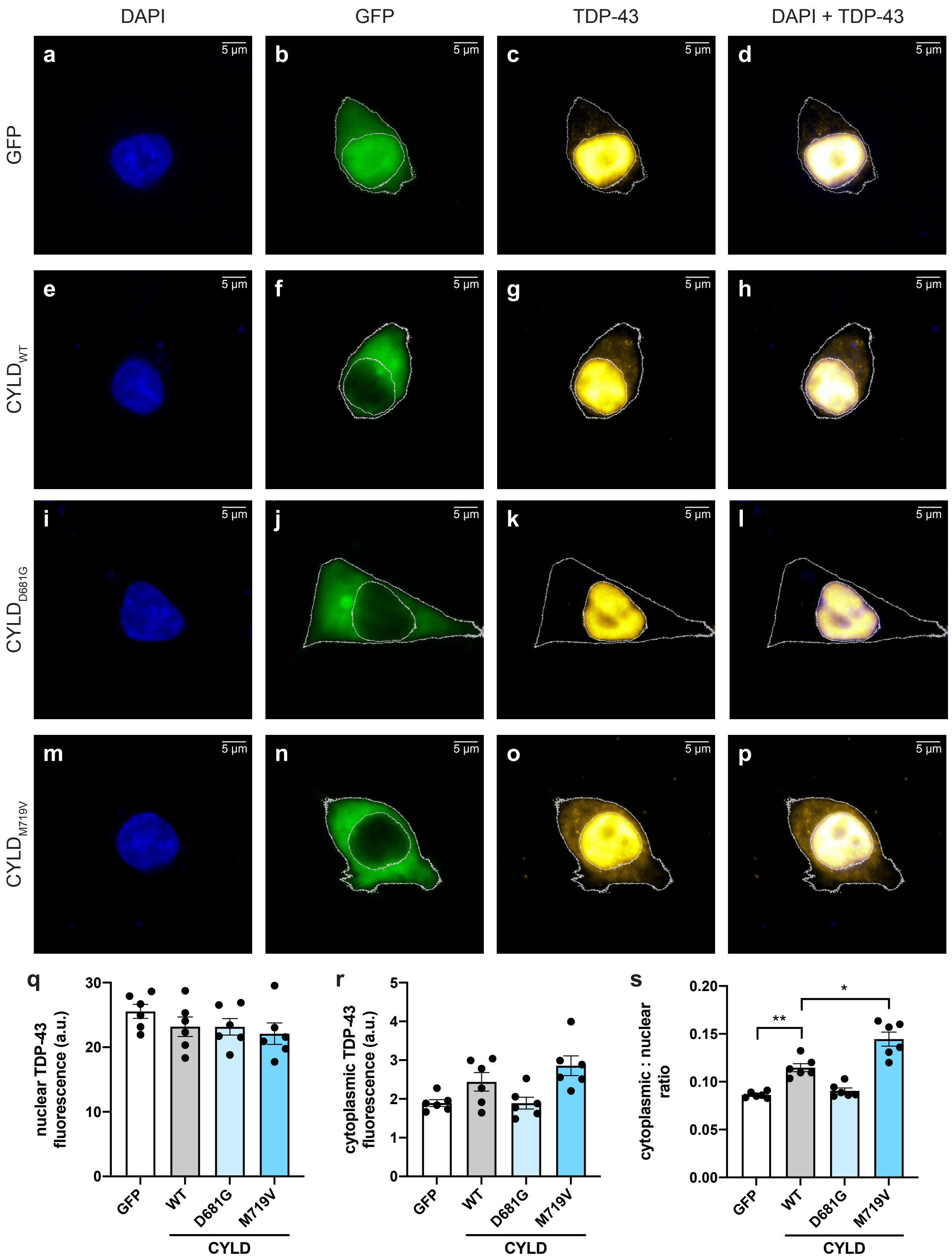

Supplementary Figure S4. Detection of endogenous TDP-43 cytoplasmic mislocalisation in cells expressing *CYLD* mutations. Representative images of HEK293 cells overexpressing (**a-d**) GFP or GFP-tagged (**e-h**) CYLD$_{WT}$, (**i-l**) CYLD$_{D681G}$ or (**m-p**) CYLD$_{M719V}$. TDP-43 was

detected by immunofluorescent staining (yellow) and nuclei were visualised with DAPI (blue). (**q**) Quantification of the fluorescence intensity of the nucleus and (**r**) cytoplasm shows no difference in TDP-43 expression. (**s**) Quantification of the cytoplasmic/nuclear ratio of exogenous TDP-43 shows a marked increase in $CYLD_{WT}$, when compared to cells expressing GFP. The cytoplasmic/nuclear ratio of TDP-43 in $CYLD_{M719V}$-expressing cells was significantly higher than $CYLD_{WT}$. Scale bars = 5 μm. Data is represented as mean ± SEM. a.u. = arbitrary units. *p<0.05; **p<0.01.
